# Patients with early-stage oropharyngeal cancer can be identified with label-free serum proteomics

**DOI:** 10.1038/s41416-018-0162-2

**Published:** 2018-07-02

**Authors:** Anna Tuhkuri, Mayank Saraswat, Antti Mäkitie, Petri Mattila, Robert Silén, Amy Dickinson, Timo Carpén, Tiialotta Tohmola, Sakari Joenväärä, Suvi Renkonen

**Affiliations:** 1 Department of Otorhinolaryngology – Head and Neck Surgery, University of Helsinki and Helsinki University Hospital, Helsinki, 00130 Finland; 2 Transplantation Laboratory, University of Helsinki, Haartmaninkatu 3, PO Box 21, Helsinki, 00014 Finland; 3 HUSLAB, Helsinki University Hospital, Helsinki, 00290 Finland; 4 Division of Ear, Nose and Throat Diseases, Department of Clinical Sciences, Intervention and Technology, Karolinska Institutet and Karolinska Hospital, Stockholm, Sweden; 5 Department of Biosciences, University of Helsinki, PO Box 65, Helsinki, 00014 Finland; 6 Department of Biosciences and Nutrition, Karolinska Institutet, Stockholm, 11382 Sweden

**Keywords:** Tumour biomarkers, Diagnostic markers

## Abstract

**Background:**

The increasing incidence of oropharyngeal squamous cell carcinoma (OPSCC) is mainly related to human papillomavirus (HPV) infection. As OPSCCs are often diagnosed at an advanced stage, mortality and morbidity remain high. There are no diagnostic biomarkers for early detection of OPSCC.

**Methods:**

Serum from 25 patients with stage I–II OPSCC, and 12 healthy controls, was studied with quantitative label-free proteomics using ultra-definition MS^E^. Statistical analyses were performed to identify the proteins most reliably distinguishing early-stage OPSCCs from controls. P16 was used as a surrogate marker for HPV. P16-positive and P16-negative tumours were analysed separately.

**Results:**

With two or more unique proteins per identification, 176 proteins were quantified. A clear separation between patients with early-stage tumours and controls was seen in principal component analysis. Latent structures discriminant analysis identified 96 proteins, most reliably differentiating OPSCC patients from controls, with 13 upregulated and 83 downregulated proteins in study cases. The set of proteins was studied further with network, pathway and protein–protein interaction analyses, and found to participate in lipid metabolism, for example.

**Conclusions:**

We found a set of serum proteins distinguishing early-stage OPSCC from healthy individuals, and suggest a protein set for further evaluation as a diagnostic biomarker panel for OPSCC.

## Introduction

The worldwide annual incidence of head and neck cancers is almost 700,000, and 380,000 patients succumb to their disease annually.^1^ Oropharyngeal squamous cell carcinoma (OPSCC) accounts for ~20% of all new head and neck cancers, and the incidence is expected to rise over the following decades.^1–3^ This increase is mainly due to the cancers related to the human papillomavirus (HPV), and particularly due to its high-risk genotype HPV-16.^2, 4^

Traditionally, the main risk factors for OPSCC have been smoking and heavy alcohol consumption.^5^ Patients diagnosed with HPV-related OPSCC tend to be younger, and the consumption of alcohol and tobacco is often lower or even absent.^6^ HPV-related tumours have a better prognosis, a lower risk of secondary malignancies and the disease responds better to (chemo)radiotherapy.^5, 7^ It is also of note that HPV-associated OPSCCs in tobacco users behave like classical tobacco-associated OPSCCs.^8^ While the de-escalation of HPV-positive OPSCC patients’ treatment is under investigation,^7^ patients with HPV-negative OPSCC still require heavy treatment and the prognosis remains poor.^6^ At the moment, the only way to improve the prognosis of patients with HPV-negative tumours would be to diagnose them earlier.

Currently, there are no diagnostic biomarkers for OPSCC to enhance its detection at an earlier stage. Brush samples, used successfully for cervical cancer screening, have been shown to be ineffective in screening HPV-positive OPSCCs, and no diagnostic biomarkers from standard bio-fluids exist.^9^ HPV vaccinations could eventually decrease the epidemic of HPV-related OPSCC; however, even if effective vaccination programmes were launched, the decrease in incidence would only be seen after a couple of decades.^10^

Protein expression levels in both tumour tissue and serum samples of patients with OPSCC have been studied, showing some alterations, compared with those of healthy controls.^11–16^ However, these studies have often been targeted to recognised proteins, based on earlier studies on other cancers. Discovery-driven mass spectrometry proteomics offers the possibility to discover novel biomarkers and pathways, as well as to associate the findings with clinical aspects.

Our objective was to compare the serum protein profiles of patients with early-stage OPSCC and of healthy controls, to promote early cancer diagnostics. For early-stage tumours, we chose stage I and stage lI tumours (eighth edition of TNM classification of malignant tumours, 2016). Protein p16, i.e. cyclin-dependent kinase inhibitor 2A, is used as a surrogate marker for HPV status at our department and also in this study. The protein was first presented for OPSCC by Klussmann et al. and is now an established immunohistological marker, widely used instead of the arduous and expensive HPV detection and typing.^17^ We analysed the serum samples in ultra-definition MS^E^ (UDMS^E^) mode. Of three data-independent data-acquisition methods available in the Synapt G2-S (MSE, high-definition MSE (HDMSE) and UDMSE), the last one was chosen as it gives the best protein coverage on the sample.^18^ Based on the proteomic changes revealed, we aimed to find a set of proteins that are possibly usable as a biomarker panel for early-stage OPSCC.

## Materials and methods

### Patients and serum samples

Serum samples from 25 patients diagnosed with stage I–II OPSCC were collected prior to treatment between the years 2012 and 2015 at the Department of Otorhinolaryngology—Head and Neck Surgery, Helsinki University Hospital, Helsinki, Finland. After collection, the samples were allowed to clot at room temperature (RT) before they were centrifuged at 4 °C (1000 × *g*) to separate serum. Sera were stored at –70 °C until all were assayed at the same time. The inclusion strategy by the TNM status was based on the eighth edition of TNM classification of malignant tumours, dividing HPV-positive and HPV-negative OPSCCs as separate entities,^8^ and protein p16 status was used as a surrogate marker for HPV. Twelve serum samples from age-matched and gender-matched control patients were received from the Finnish Red Cross Blood Service.

Written informed consent was obtained from all patients. The study plan was approved by the institutional Research Ethics Board at the Helsinki University Hospital (DNr. 51/13/03/02/2013).

### Reagents

Reagents for serum pre-processing, Pierce Swell Gel Blue Albumin Removal Discs, Pierce Centrifuge columns and Pierce C18 Spin Columns, were acquired from Thermo Scientific (Rockform, IL, USA), solvents and high-purity HPLC reagents from Waters (Milford, MA, USA) and other reagents from Sigma-Aldrich (St Louis, MO, USA).

### Serum treatment and protein digestion

The workflow has been described previously in detail.^19^ In brief, the samples were thawed, and after the depletion of the top 12 proteins with Pierce Top 12 protein depletion columns, the protein concentration was measured by a bicinchonic acid assay kit (Pierce, Thermo Scientific, Rockform, IL, USA) for the total protein concentration. Top 12 protein-depleted serum samples corresponding to 350 µg of total protein were dried in a speed vacuum (Savant, Thermofisher), and then dissolved in 6 M urea and 100 mM Tris-HCl (pH 7.4). Reduction of disulphide bonds was performed with 10 mM of dithiothreitol (DTT) for 60 min at RT, and thereafter 30 mM iodoacetamide was used for alkylating the proteins for 60 min in the dark at RT. Protein digestion was performed with trypsin (Promega, Madison, WI) for 18 h at +37 °C after the consumption of excess iodoacetamide by adding DTT again (30 mM DTT, 60 min at RT). Samples were diluted 1:10 with high-purity Milli-Q water (Millipore, Billerica, MA, USA) before addition of trypsin. Finally, the samples were purified in C18 spin columns, and dried in a speed vacuum and dissolved in 0.1% formic acid containing 12.5 fmol Hi3 peptide mixture (Waters) per µl. All of the procedures described were performed according to the manufacturer’s instructions, wherever applicable.

### Liquid chromatography—ultra-definition MS^E^

Four-microlitre samples corresponding to 1.4 µg of total protein were injected to the ultra-performance liquid chromatography (UPLC) system (Waters Corporation, Billerica, MA, USA).^18^ TRIZAIC nanoTile 88-µm × 100-mm HSS-T3u wTRAP was applied as a separating device before mass spectrometry (MS). After loading and trapping, the samples were washed for 2 min at 8.0 µl/min with 1% buffer B. The analytical gradient was used as follows: 0–1 min 1% B; at 2 min 5% B; at 65 min 30% B; at 78 min 50% B; at 80 min 85% B; at 83 min 85% B; at 84 min 1% B and at 90 min 1% B with 450 nl/min. Buffer A consists of 0.1% formic acid in water and buffer B consists of 0.1% formic acid in acetonitrile (Sigma-Aldrich).

The data were acquired with UDMS^E^ with Synapt G2-S UDMS (Waters Corporation) including ion mobility spectroscopy (IMS). The data range was 100–2000*m/z*, scan time 1 s, IMS wave velocity 650 ms^−1^ and collision energy ramped in trap between 20 and 60 V. Calibration was performed by Glu1-fibrinopeptide B MS2 fragments and Glu1-fibrinopeptide B precursor ion, used during the acquisitions as a lock mass. In total, 10% of the samples were acquired as triplicates to validate the results, and further analysis was conducted with Progenesis QI for Proteomics software (Nonlinear Dynamics, Newcastle, UK) (Supplement S2—triplets).

The mass spectrometry proteomics data have been deposited into the ProteomeXchange Consortium via the PRIDE partner repository with the data set identifier PXD008445.^20^

### Data analysis

The data analysis was described previously in detail.^21^ Briefly, Progenesis QI for proteomics software (Version 3, Nonlinear Dynamics) was used for processing raw files. Peptide identification was run with Uniprot human FASTA sequences (UniprotKB Release 2015_09, 20205 sequence entries), and label-free protein quantification was performed with the Hi-N method (Protein Lynx Global Server).^22^ The samples were spiked with 12.5 fmol/µl of CLPB_ECOLI (P63285, ClpB protein) peptides (Hi3 *Escherichia Coli* Standard, Waters).

The peptide identification parameters were fixed modification of cysteine (carbamidomethyl) and variable modification of methionine (oxidation). The peptide error tolerance was set to a maximum of 10 ppm, the false-discovery rate was limited to less than 2% and default values (in Progenesis QI for Proteomics) were used for the rest of the parameters.

The quantified proteins in all comparisons were compared by ANOVA on a protein-by-protein basis and their expression levels were considered significantly different if the ANOVA p value was <0.05. Principal component analysis (PCA), offering the visualisation of the main axes of variation in the data groups, was performed by Progenesis QI for proteomics. Processing the Progenesis QI data with EZinfo 3.0 software (a statistical tool released in December 2014, Umetrics, Sweden), supervised OPLS–DA modelling was performed. With a *p*(corr) cut-off of ± 0.80, a variance versus correlation plot (S-plot) and a list of S-plot proteins was generated from OPLS–DA data.

### Protein–protein interactions, pathways and networks

STRING 10.5 database illustrates known and predicted protein–protein interactions (PPI),^23^ and was used for PPI analyses, giving a sophisticated view of possible and known interactions between proteins. PPI analyses were conducted to filter the S-plot proteins and project them to connected pathways and/or co-expression. Medium stringency was used for inferring the networks from protein lists on the STRING DB and textmining was excluded as a setting.

The network and canonical pathway overrepresentation analyses were conducted through the use of Ingenuity pathway analysis (IPA; QIAGEN Inc., https://www.qiagenbioinformatics.com/products/ingenuity-pathway-analysis) with default parameters to identify which networks and pathways were most enriched in our protein list.^24^ IPA networks differ from PPIs in their way of connecting proteins. In addition to the proteins actually present, they combine the information about possible connector proteins (not present in the user-supplied list). This allows another way of finding the networks the proteins are enriched into. IPA analyses were conducted on the proteins with the ANOVA *p* value < 0.05 and S-plot proteins were then separately matched to the proteins in enriched networks.

## Results

### Metadata and workflow

Twenty-five serum samples from patients with stage I and stage II OPSCC, together with 12 samples from healthy controls were studied. Of the 25 patients with stage I–II tumours, 12 had p16-positive and 13 had p16-negative tumours.

**Fig. 1:**
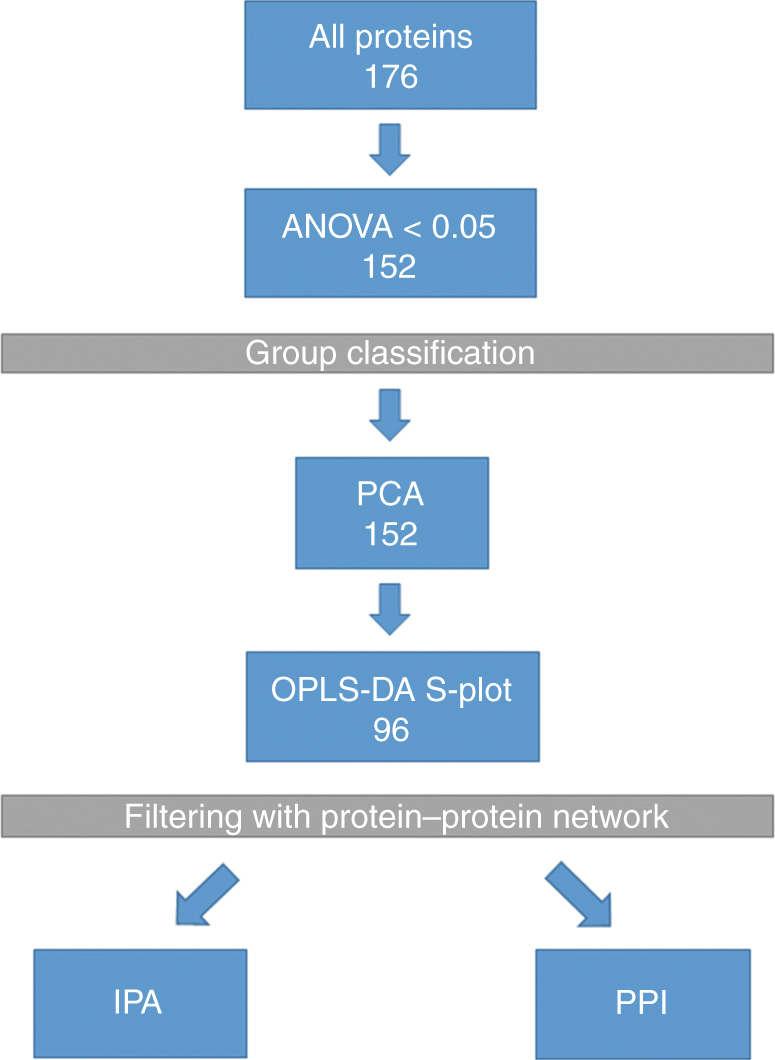
Data analysis workflow. Protein quantification data were from ultra-definition MSE, and proteins with two or more unique peptides were approved for identification. ANOVA cut-off of 0.05 was used. PCA: principal component analysis is used to visualise the variation between groups. OPLS–DA: latent structures discriminant analysis brings data for the S-plot for an efficient comparison of protein expression profiles. PPI: protein–protein interaction network gives the known and predicted functional and physical associations between single proteins in the S-plot. IPA: Ingenuity pathway analysis is an analysis tool revealing pathways and potential networks associated with the given data

The tumour localisation was tonsil in 15 (60%) of the 25 patients, base of the tongue in 8 (32%), the soft palate in 8 (32%) and posterior wall of the oropharynx in 1 (4%). Sixty percent of the patients were male and 40% were female. The age of the patients varied from 36 to 78 years with the median age being 60.85 (average 60.92). More detailed clinical parameters are provided in Supplementary Table 1. The data analysis workflow is presented in Fig. 1.

#### All early-stage OPSCCs versus controls

##### Protein identification and PCA

**Fig. 2:**
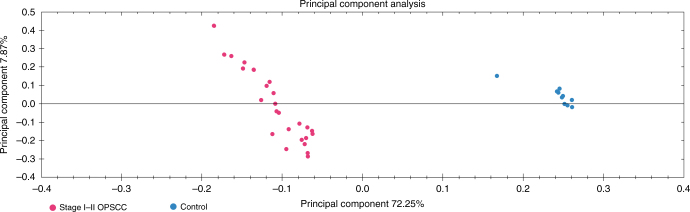
Principal component analysis using serum protein expression data of early-stage OPSCC versus controls (two or more unique peptides, ANOVA *p* value < 0.05). Early-stage tumour samples are marked with red and controls are marked with blue

With the criterion of two or more unique peptides per protein identification, 176 proteins were quantified from all cases and controls were analysed. The identified proteins were compared by ANOVA on a protein-to-protein basis. With the ANOVA cut-off of 0.05, 152 proteins with two or more unique peptides were quantified (Supplementary Table 2). Based on serum protein expression levels of patients with early-stage OPSCC and healthy controls, the two groups were found to be separated in PCA (Fig. 2).

##### OPLS–DA

**Fig. 3:**
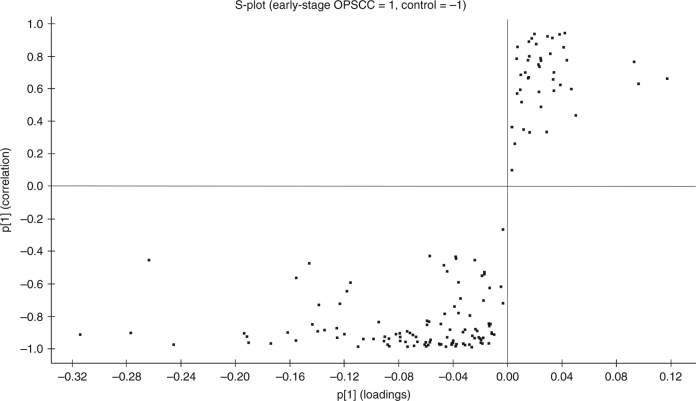
S-plot obtained from OPLS–DA regression analysis of the serum protein expressions in early-stage OPSCCs versus controls (*p*(corr) ± 0.80). The proteins were downregulated in tumour patients’ serum at the upper-right corner and upregulated on the lower left

As another group classification method, OPLS–DA modelling was performed, and an S-plot was generated, presenting 96 proteins that most reliably distinguished patients from controls (Fig. 3). These proteins passed the *p*(corr) cut-off of ± 0.80 and were thus considered significantly different (Table 1). Of the 96 proteins, 13 were expressed in higher levels in early-stage OPSCCs when compared to controls, and the remaining 83 proteins had lower levels in cases compared with controls.

**Table 1:** S-plot proteins obtained from OPLS–DA regression analysis (*p*(corr) ± 0.80), of all stage I–II OPSCCs versus controls

Primary accession	Peptide count	Unique peptides	Confidence score	ANOVA (p)	Max fold change	Highest mean	Lowest mean	Protein name	Gene name	p[1]	p(corr)[1]
P19105	2	2	10.19	0	184.10	Stage I–II OPSCC	Control	Myosin regulatory light chain 12A	MYL12A	–0.04	–0.95
P01034	2	2	12.07	0	Infinity	Stage I–II OPSCC	Control	Cystatin-C	CST3	–0.02	–0.95
Q8N2Z9	3	3	15.95	0	14.92	Stage I–II OPSCC	Control	Centromere protein S	APITD1	–0.04	–0.94
Q09013;M0QX08	4	3	35.22	0	9.23	Stage I–II OPSCC	Control	Myotonin-protein kinase	DMPK	–0.03	–0.92
P15428	2	2	11.07	0	Infinity	Stage I–II OPSCC	Control	15-hydroxyprostaglandin dehydrogenase [NAD(+)]	HPGD	–0.02	–0.92
Q8N3Y7	5	3	33.98	1.12E−10	10.36	Stage I–II OPSCC	Control	Epidermal retinol dehydrogenase 2	SDR16C5	–0.03	–0.91
P36980	12	4	74.12	0	8.55	Stage I–II OPSCC	Control	Complement factor H-related protein 2	CFHR2	–0.02	–0.89
A0A0B4J2B5	3	2	10.40	0	35.11	Stage I–II OPSCC	Control	Protein IGHV3R16-9 (fragment)	IGHV3R16-9	–0.01	–0.87
P48736	6	4	34.80	2.00E−14	8.29	Stage I–II OPSCC	Control	Phosphatidylinositol 4,5-bisphosphate 3-kinase catalytic subunit gamma isoform	PIK3CG	–0.04	–0.86
Q3MJ40	3	2	24.36	1.11E−16	10.49	Stage I–II OPSCC	Control	Coiled-coil domain-containing protein 144B	CCDC144B	–0.02	–0.85
O94887	7	3	36.29	3.27E−07	3.80	Stage I–II OPSCC	Control	FERM, RhoGEF and pleckstrin domain-containing protein 2	FARP2	–0.01	–0.81
Q9C091	8	6	43.13	0	9.39	Stage I–II OPSCC	Control	GREB1-like protein	GREB1L	–0.03	–0.81
A0A1B0GUA9	2	2	11.10	0	Infinity	Stage I–II OPSCC	Control	Uncharacterised protein	N/A	–0.02	–0.80
P07357	39	37	309.89	6.18E−07	1.69	Control	Stage I–II OPSCC	Complement component C8 alpha chain	C8A	0.06	0.80
P05452	19	17	183.04	1.11E−07	1.52	Control	Stage I–II OPSCC	Tetranectin	CLEC3B	0.04	0.80
P02753	48	42	241.90	2.55E−07	2.03	Control	Stage I–II OPSCC	Retinol-binding protein 4	RBP4	0.09	0.81
P07996	26	24	196.30	1.55E−09	3.15	Control	Stage I–II OPSCC	Thrombospondin-1	THBS1	0.06	0.81
P05543	20	18	142.13	3.40E−07	2.40	Control	Stage I–II OPSCC	Thyroxine-binding globulin	SERPINA7	0.05	0.82
P01019	81	76	374.07	7.15E−09	2.58	Control	Stage I–II OPSCC	Angiotensinogen	AGT	0.14	0.84
Q6PI47	3	3	22.91	2.59E−08	2.44	Control	Stage I–II OPSCC	BTB/PZ domain-containing protein KCTD18	KCTD18	0.06	0.85
Q7Z6K3	3	3	29.13	6.27E−09	6.48	Control	Stage I–II OPSCC	Protein prenyltransferase alpha subunit repeat-containing protein 1	PTAR1	0.03	0.85
P00734	106	90	686.35	1.45E−09	1.82	Control	Stage I–II OPSCC	Prothrombin	F2	0.13	0.85
O14791	11	6	64.95	2.10E−08	3.10	Control	Stage I–II OPSCC	Apolipoprotein L1	APL1	0.01	0.85
P03952	53	44	476.60	4.74E−10	1.63	Control	Stage I–II OPSCC	Plasma kallikrein	KLKB1	0.07	0.85
P42336	9	3	45.78	8.43E−09	2.46	Control	Stage I–II OPSCC	Phosphatidylinositol 4,5-bisphosphate 3-kinase catalytic subunit alpha isoform	PIK3CA	0.01	0.86
P43652	108	90	739.29	3.46E−09	1.88	Control	Stage I–II OPSCC	Afamin	AFM	0.12	0.86
Q5VU65	5	3	22.62	1.67E−10	6.46	Control	Stage I–II OPSCC	Nuclear pore membrane glycoprotein 210-like	NUP210L	0.01	0.87
P00740	16	14	113.09	7.90E−09	3.73	Control	Stage I–II OPSCC	Coagulation factor IX	F9	0.04	0.87
P04196	62	58	332.49	5.30E−09	2.62	Control	Stage I–II OPSCC	Histidine-rich glycoprotein	HRG	0.14	0.87
P43251	7	6	49.04	1.04E−07	3.04	Control	Stage I–II OPSCC	Biotinidase	BTD	0.02	0.87
P04004	83	65	503.49	1.96E−10	2.03	Control	Stage I–II OPSCC	Vitronectin	VTN	0.16	0.87
Q8IVH4	3	3	26.12	9.95E−06	3.33	Control	Stage I–II OPSCC	Methylmalonic aciduria type-A protein, mitochondrial	MMAA	0.02	0.88
P08185	27	23	212.28	7.43E−10	2.33	Control	Stage I–II OPSCC	Corticosteroid-binding globulin	SERPINA6	0.07	0.88
P02749	124	109	472.25	1.30E−10	2.15	Control	Stage I–II OPSCC	Beta-2-glycoprotein 1	APH	0.27	0.88
Q14624	138	123	984.03	4.93E−10	1.91	Control	Stage I–II OPSCC	Inter-alpha-trypsin inhibitor heavy chain H4	ITIH4	0.19	0.89
P08697	38	36	326.03	4.46E−11	1.95	Control	Stage I–II OPSCC	Alpha-2-antiplasmin	SERPINF2	0.08	0.89
P00915	3	2	16.19	0	Infinity	Control	Stage I–II OPSCC	Carbonic anhydrase 1	CA1	0.01	0.89
Q9UK55	8	7	43.36	0	8.09	Control	Stage I–II OPSCC	Protein Z-dependent protease inhibitor	SERPINA10	0.08	0.89
Q15102	2	2	10.00	0	1344.35	Control	Stage I–II OPSCC	Platelet-activating factor acetylhydrolase IB subunit gamma	PAFAH1B3	0.01	0.89
P20851	10	6	78.00	0	45.67	Control	Stage I–II OPSCC	C4b-binding protein beta chain	C4BPB	0.03	0.89
P05090	24	23	200.64	2.42E−14	2.69	Control	Stage I–II OPSCC	Apolipoprotein D	APD	0.09	0.90
P02774	206	181	1148.20	2.81E−11	1.79	Control	Stage I–II OPSCC	Vitamin D-binding protein	GC	0.31	0.90
P19652	31	20	133.66	1.44E−15	6.38	Control	Stage I–II OPSCC	Alpha-1-acid glycoprotein 2	RM2	0.07	0.90
Q52LA3	4	3	26.69	6.92E−09	19.54	Control	Stage I–II OPSCC	Protein lin-52 homologue	LIN52	0.02	0.90
P01591	3	2	21.76	0	20.46	Control	Stage I–II OPSCC	Immunoglobulin J chain	JCHAIN	0.01	0.91
P02748	80	70	553.63	3.29E−11	2.21	Control	Stage I–II OPSCC	Complement component C9	C9	0.12	0.91
P41222	2	2	11.91	0	13700.40	Control	Stage I–II OPSCC	Prostaglandin-H2 D-isomerase	PTGDS	0.02	0.91
P00747;Q02325;Q15195	154	132	1214.37	2.25E−11	2.00	Control	Stage I–II OPSCC	Plasminogen	PLG	0.19	0.91
Q9NZP8	3	2	19.85	2.79E−07	194.65	Control	Stage I–II OPSCC	Complement C1r subcomponent-like protein	C1RL	0.02	0.92
P80108	7	7	57.61	4.83E−11	3.28	Control	Stage I–II OPSCC	Phosphatidylinositol–glycan-specific phospholipase D	GPLD1	0.03	0.92
P0C0L5	394	16	2558.66	2.67E−08	6.78	Control	Stage I–II OPSCC	Complement C4-B	C4B	0.11	0.93
P01031	131	116	977.48	3.05E−13	2.28	Control	Stage I–II OPSCC	Complement C5	C5	0.13	0.93
Q9Y2H2	7	5	31.34	1.11E−16	14.32	Control	Stage I–II OPSCC	Phosphatidylinositide phosphatase SAC2	INPP5F	0.08	0.93
P09871	50	42	381.14	1.18E−13	2.02	Control	Stage I–II OPSCC	Complement C1s subcomponent	C1S	0.08	0.93
Q96KN2	18	16	138.56	0	3.87	Control	Stage I–II OPSCC	Beta-Ala-His dipeptidase	CNDP1	0.05	0.94
P0C0L4	393	14	2503.26	9.99E−16	49.09	Control	Stage I–II OPSCC	Complement C4-A	C4A	0.19	0.94
P08294	4	3	29.94	0	36.98	Control	Stage I–II OPSCC	Extracellular superoxide dismutase [Cu–Zn]	SD3	0.02	0.94
Q7LC44	3	2	22.46	7.34E−13	316.16	Control	Stage I–II OPSCC	Activity-regulated cytoskeleton-associated protein	ARC	0.02	0.94
O95445	12	11	85.78	1.55E−15	3.46	Control	Stage I–II OPSCC	Apolipoprotein M	APM	0.06	0.94
P02649	70	62	492.63	3.32E−14	3.40	Control	Stage I–II OPSCC	Apolipoprotein E	APE	0.16	0.94
P27918	10	8	82.75	0	3.61	Control	Stage I–II OPSCC	Properdin	CFP	0.04	0.94
P22352	23	20	129.93	1.11E−16	3.73	Control	Stage I–II OPSCC	Glutathione peroxidase 3	GPX3	0.07	0.95
P07358	60	51	461.28	2.22E−16	2.42	Control	Stage I–II OPSCC	Complement component C8 beta chain	C8B	0.07	0.95
P22792	28	25	266.69	3.08E−14	2.57	Control	Stage I–II OPSCC	Carboxypeptidase N subunit 2	CPN2	0.09	0.95
Q08629	4	2	34.99	1.01E−08	31.43	Control	Stage I–II OPSCC	Testican-1	SPCK1	0.03	0.95
Q04756	8	7	54.20	0	228.12	Control	Stage I–II OPSCC	Hepatocyte growth factor activator	HGFAC	0.04	0.95
P00736	54	43	423.82	2.89E−15	2.58	Control	Stage I–II OPSCC	Complement C1r subcomponent	C1R	0.10	0.95
O75636	8	7	88.24	0	24.29	Control	Stage I–II OPSCC	Ficolin-3	FCN3	0.07	0.95
A0A096LPE2;P35542	35	15	202.65	0	6.02	Control	Stage I–II OPSCC	Protein SAA2–SAA4	SAA2–SAA4	0.06	0.96
P06681	31	8	254.03	6.01E−13	4.48	Control	Stage I–II OPSCC	Complement C2	C2	0.04	0.96
P19827	126	107	848.22	3.33E−16	3.17	Control	Stage I–II OPSCC	Inter-alpha-trypsin inhibitor heavy chain H1	ITIH1	0.24	0.96
P15169	10	9	93.43	0	9.89	Control	Stage I–II OPSCC	Carboxypeptidase N catalytic chain	CPN1	0.06	0.96
Q13790	3	2	27.72	1.10E−13	22.60	Control	Stage I–II OPSCC	Apolipoprotein F	APF	0.02	0.96
P07360	19	15	166.71	2.64E−14	2.87	Control	Stage I–II OPSCC	Complement component C8 gamma chain	C8G	0.09	0.96
P02747	8	8	60.45	6.85E−14	4.08	Control	Stage I–II OPSCC	Complement C1q subcomponent subunit C	C1QC	0.05	0.96
P04070	4	2	23.64	1.11E−15	4.71	Control	Stage I–II OPSCC	Vitamin K-dependent protein C	PRC	0.01	0.96
P13645	16	15	115.79	0	6.25	Control	Stage I–II OPSCC	Keratin, type-I cytoskeletal 10	KRT10	0.06	0.96
P00748	3	3	18.59	0	679.60	Control	Stage I–II OPSCC	Coagulation factor XII	F12	0.03	0.97
P04003	82	74	628.10	1.11E−16	2.84	Control	Stage I–II OPSCC	C4b-binding protein alpha chain	C4BPA	0.18	0.97
Q7Z794	6	3	44.34	0	48569.15	Control	Stage I–II OPSCC	Keratin, type-II cytoskeletal 1b	KRT77	0.05	0.97
P07225	32	27	244.61	0	3.08	Control	Stage I–II OPSCC	Vitamin K-dependent protein S	PRS1	0.09	0.97
P06276	7	6	56.00	0	11.44	Control	Stage I–II OPSCC	Cholinesterase	BCHE	0.04	0.97
P27482	2	2	11.92	1.34E−07	25.89	Control	Stage I–II OPSCC	Calmodulin-like protein 3	CALML3	0.02	0.97
P49908	9	7	46.07	0	41.43	Control	Stage I–II OPSCC	Selenoprotein P	SELENP	0.04	0.98
O00750	11	4	59.49	0	4.02	Control	Stage I–II OPSCC	Phosphatidylinositol 4-phosphate 3-kinase C2 domain-containing subunit beta	PIK3C2B	0.05	0.98
P08571	6	6	51.89	0	6.40	Control	Stage I–II OPSCC	Monocyte differentiation antigen CD14	CD14	0.03	0.98
Q9UGM5	8	8	54.43	1.08E−14	13.76	Control	Stage I–II OPSCC	Fetuin-B	FETUB	0.03	0.98
P35858	32	29	277.24	0	11.46	Control	Stage I–II OPSCC	Insulin-like growth factor-binding protein complex acid-labile subunit	IGFALS	0.11	0.98
O75882	22	20	150.98	0	15.94	Control	Stage I–II OPSCC	Attractin	ATRN	0.06	0.98
P60709;P63261;P62736;P63267;P68032;P68133	6	3	45.19	0	Infinity	Control	Stage I–II OPSCC	Actin, cytoplasmic 1	ACTB	0.05	0.99
Q96IY4	5	5	35.72	0	18.33	Control	Stage I–II OPSCC	Carboxypeptidase B2	CPB2	0.04	0.99
P17936	8	6	58.46	0	21.95	Control	Stage I–II OPSCC	Insulin-like growth factor-binding protein 3	IGFBP3	0.03	0.99
P06396	76	3	631.02	1.11E−16	407.24	Control	Stage I–II OPSCC	Gelsolin	GSN	0.06	0.99
P02746	12	11	106.18	0	8.16	Control	Stage I–II OPSCC	Complement C1q subcomponent subunit B	C1QB	0.07	0.99
Q14520	19	17	146.18	0	7.40	Control	Stage I–II OPSCC	Hyaluronan-binding protein 2	HABP2	0.07	0.99
Q5T6V5	5	4	38.73	0	64.40	Control	Stage I–II OPSCC	UPF0553 protein C9orf64	C9orf64	0.09	0.99

##### Protein–protein interactions

To further study our set of S-plot proteins and to try to identify the most relevant proteins, protein–protein interaction (PPI) webs were created using the STRING 10.5 database. Proteins with the most interactions, with connections to other proteins ranging from 9 to 16, were prothrombin (F2), plasminogen (PLG), alpha-2-antiplasmin (SERPINF2), histidine-rich glycoprotein (HRG), beta-2-glycoprotein 1 (APOH), carboxypeptidase B2 (CPB2), inter-alpha-trypsin inhibitor heavy chain H4 (ITIH4) and complement C2, C5, C4-A and C4-B (C2, C5, C4A and C4B).

**Fig. 4:**
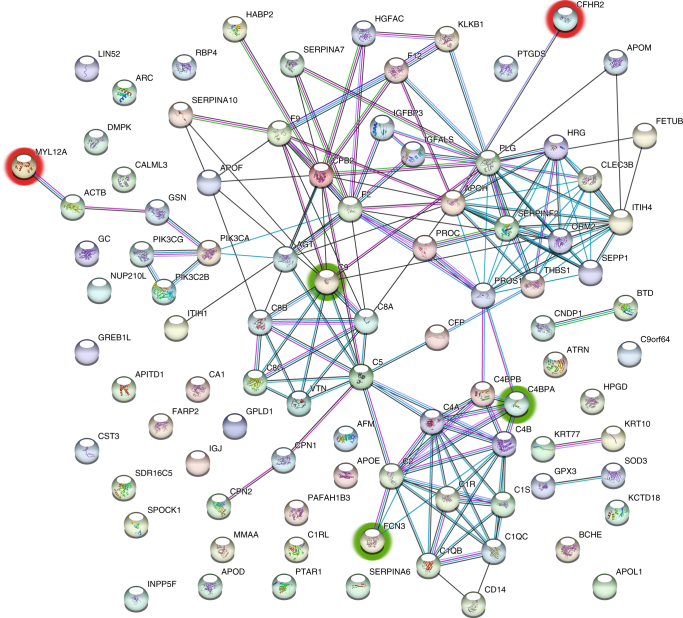
PPI network of S-plot proteins (*p*(corr) ± 0.80) manifesting in the stage I–II OPSCC. The five serum proteins discussed in the article as possible biomarkers for early-stage OPSCC and suggested for further screening are circled: CFHR2 and MYL12A, upregulated in the tumour patients’ serum are circled with red, and the downregulated C9, FCN3 and C4BPA are circled with green

According to the UNIPROT database,^25^ these proteins seemed to be associated with complement activation (early and late), extracellular matrix remodelling and lipid metabolism, for example. PPIs of the S-plot proteins are shown in Fig. 4.

##### Pathways and networks

**Fig. 5:**
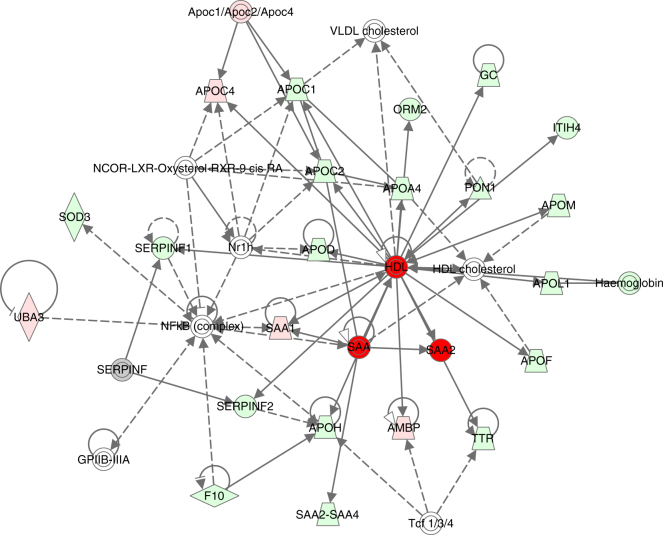
IPA network 2. Lipid metabolism, molecular transport and small-molecule biochemistry with a score of 45. See text for details

The top six IPA networks where the identified proteins were most enriched were 1. developmental disorder, hereditary disorder and immunological disease; 2. lipid metabolism, molecular transport and small-molecule biochemistry; 3. humoral immune response, inflammatory response, haematological system development and function; 4. cardiovascular disease, organismal injury and abnormalities and tissue morphology; 5. hereditary disorder, ophthalmic disease, organismal injury and abnormalities and 6. cell morphology, cellular development, cellular assembly and organisation. The score of the top six IPA networks ranged from 21 to 45. There were 13–23 proteins with the ANOVA p value < 0.05 participating in each of the networks and the total amount of focus molecules was 108. Of these, 46 were S-plot proteins (*p*(corr) ± 0.80). The network linked with lipid metabolism, containing 14 S-plot proteins, is illustrated in Fig. 5. The other five networks are presented in Supplementary Table 3. Altogether, among the S-plot proteins present in the top six IPA networks, four were upregulated in cases versus controls: complement factor H-related protein 2 (CFHR2), GREB1-like protein (GREB1L), myosin regulatory light chain 12A (MYL12A) and myotonin-protein kinase (DMPK). CFHR2 and MYL12A were also found to be binding in the PPI clusters. The remaining 42 S-plot proteins presented in the top three IPA networks were downregulated in cases versus controls, and the majority of these were also present in the PPI clusters.

In the canonical pathway analyses conducted with IPA, acute phase response signalling, LXR/RXR activation, FXR/RXR activation and the complement system were among the highest enriched pathways. The top canonical pathways are shown in Supplementary Figure 1.

#### Comparison between P16-negative and P16-positive tumours

##### Protein identification and PCA

In a comparison between p16-negative early-stage tumours and controls, 148 proteins were found with different expression levels in the serum samples. In the case of p16-positive early-stage tumours, the number was 152. When comparing the p16-negative and p16-positive groups with each other, 24 proteins were differently expressed. The protein identification tables are presented in Supplementary Table 2 and PCAs are presented in Supplementary Figures 4–6.

##### OPLS–DA

In the comparison between patients with early-stage p16-negative OPSCC and healthy controls, 103 proteins were presented in the S-plot (*p*(corr) ± 0.80), and 104 proteins were presented in the comparison of p16-positive tumours versus controls, respectively (Supplementary Figures 7 and 8). Of these, 96 were common between the two groups, shown in a Venn diagram (Supplementary Figure 9). It is of note, though, that the fold changes of the S-plot proteins were not identical in the two groups. The lists of S-plot proteins in the two comparisons are presented in Supplementary Table 4.

##### Protein–protein interactions

PPI networks of p16-positive and p16-negative groups were also studied separately, and the results showed great consistency with those from all early-stage OPSCC samples combined. Minor differences between p16-positive and p16-negative groups were detected, for example, there were slight differences in the protein interactions in the coagulation pathway. The PPI networks are shown in Supplementary Figures 10 and 11.

##### Pathways and networks

In the network analysis conducted with IPA, most networks were represented in both p16-positive and p16-negative patients’ data. Some differences were found, for example, haematological disease, haematological system development and function and organismal functions were a network solely enriched in the p16-positive group. The results are shown in Supplementary Table 3. In canonical pathway analyses, the top enriched pathways were also almost identical between p16-positive and p16-negative groups with some differences in their order (Supplementary Figures 2 and 3).

## Discussion

The mortality of OPSCC ranges from 19 to 86%, the main predictive markers being tumour stage and HPV status.^8, 26^ At the moment, most tumours are diagnosed at an advanced stage, and thus the best way to improve their prognosis would be to diagnose them at an earlier stage.^27^ Currently, there are no known biomarkers to detect OPSCCs before clinical signs exist. When diagnosed, tumours are either visible or cause clinical symptoms, for example, dysphagia, pain etc.^27^ Discovering serum proteins that distinguish patients with early-stage cancer from healthy controls would be of great value from a diagnostic point of view. In order to identify possible proteins to be used as such biomarkers, we analysed serum samples of 25 patients diagnosed with stage I–II OPSCC and 12 healthy controls. Altogether, 176 serum proteins were reliably quantified, and the expression profiles of OPSCC patients differed clearly from those of healthy controls.

The discovery-driven nature of mass spectrometry-based analysis offers a unique chance to discover proteins and pathways that have not previously been studied in OPSCC. In previous serological studies, an association between serum antibodies towards HPV-16 early (E) antigens and HPV-positive OPSCC has been described, and these E antibodies have been studied as potential diagnostic biomarkers for HPV-related OPSCC. Recently, seropositivity for E6 antibodies was described as a highly sensitive (96%) and specific (98%) marker for HPV-positive OPSCC.^12^ However, in this study lacking a control group, the majority of patients had advanced stage tumours: there were 134 patients with stage lV tumours and 80 patients with stage I–III tumours. In addition, another study presenting an algorithm incorporating information about multiple E antibodies with a high sensitivity (83%) and specificity (99%) has been conducted for the detection of HPV-related OPSCC.^11^ In that study, age-matched and sex-matched healthy individuals served as healthy controls. However, analyses were made on patients with tumours of all stages and only a few represented early-stage tumours. Thus, the clinical use of the E antigens still remains an open question, as there is no information about their usability in early diagnostics, for example. In addition to E antigens, serum levels of matrix metalloproteinases (MMP1, 2 and 9) have been studied in oropharyngeal, laryngeal and hypopharyngeal carcinoma by Kalfert et al. and found not to serve as suitable prognostic tumour markers in these cancers.^14^ MMP1 expression was described as being significantly influenced by smoking and p16 expression. There was no control group in the study. Also, serum levels of IL-10, TNF-α, TGF-β, VEGF, Cyfra21-1, SCCAg, ferritin, CEA, CA19-9 and AFP have been studied in oral and oropharyngeal carcinoma patients.^15, 16^ In summary, until now, serological studies have not elucidated any biomarkers that will allow detection of oropharyngeal tumours at an early stage.

The OPLS–DA modelling generates a list of the most significant proteins in terms of group separation (S-plot proteins). This level of discrimination is difficult to obtain using other statistical methods. Statistically significant differences in expression of serum proteins between patients with early-stage OPSCC, when compared with controls, included 13 upregulated and 83 downregulated proteins. Of these, IPA networks and PPI analyses revealed interesting clusters of these proteins acting together. In the PPI network of the S-plot proteins of early-stage OPSCCs versus controls, examples of the pathways and biological processes visualised were complement activation (early and late), extracellular matrix remodelling, angiogenesis and possible tumour growth. Among the proteins with most interactions were complement C5, C4-A and C4-B (C5, C4A and C4B), prothrombin (F2), plasminogen (PLG), carboxypeptidase B2 (CPB2), alpha-2-antiplasmin (SERPINF2), histidine-rich glycoprotein (HRG) and insulin-like growth factor-associated proteins (IGFBP3, IGFALS). The complement cascade is one of the most studied biological processes in cancers.^28^ Dysregulated complement activation in the tumour microenvironment has been recently linked with increased inflammation and thus suppression of antitumour immune responses, leading to tumour cell proliferation, migration and invasive potential.^29^ The decrease of the plasmic complement C4-A has previously been described by Koifman et al. and Ornellas et al. in HPV-positive squamous cell carcinoma of the penis.^30, 31^ Also, genetic deficiency of the complement isoforms C4A or C4B may predict improved survival of metastatic renal cell carcinoma.^32^ In our study, serum levels of complement C4-A were lower in comparison with controls.

A common approach to biomarker signature discovery for any given group of patient samples is to perform a classification analysis such as the one we have done (OPLS–DA). However, by using different approaches to discovery, different molecules that differentiate the disease can be found. Moreover, the biological interpretation is often difficult due to the complicated nature of how gene/protein signatures are found, including the lack of causal relationships between protein expression and disease. The two aforementioned shortcomings are currently preventing biomarkers from becoming standard clinical tools. To circumvent these problems, network-based approaches have been proposed to be integrated with feature-selection algorithms.^33^ These network-based approaches include protein–protein interactions, canonical pathways and Gene Ontology annotations, which can help interpret the feature selection for various purposes including biomarker discovery.^34^ However, different approaches to these network-based methods lead to slightly different results, such as those employed by STRING DB or IPA.^35^ The methodology in the present work was chosen according to what has been suggested by deep analysis of common network-building software modules, i.e. that at least two different methods should be used for the purpose of network inference.^35^ To be able to filter our protein set, and to further identify a potential panel of proteins to serve as a diagnostic panel, IPA network analysis was conducted. There were six networks considered significant, having a score of 21 or more and at least 13 focus proteins.^36^ The first and third of the top six IPA networks with the best scores and focus molecules were developmental disorder, hereditary disorder and immunological disease and humoral immune response, inflammatory response, haematological system development and function. These networks were associated with complement activation, thus being consistent with the data received from PPI analyses. Proteins found in the second network, lipid metabolism, molecular transport and small-molecule biochemistry, were associated with lipoprotein metabolism and lipid digestion, mobilisation and transport. Most solid tumours tend to get hypoxic and are thus acidic.^37^ This causes tumour cells to increase their uptake of apolipoproteins, handle fatty acids more rapidly and enhance their cholesterol biosynthesis.^37^ These functions have been shown to have a big influence on tumour cell growth.^38^ Alterations of serum levels of apolipoproteins have previously been reported to be associated with breast, lung and colorectal cancers.^39^ In our material, most of the apolipoproteins participating in the networks were downregulated in the OPSCC serum compared to controls, except for apolipoprotein C-IV (APOC4) that was upregulated. This seems logical considering the increased uptake of apolipoproteins by tumour cells.

Two S-plot proteins, CFHR2 and MYL12A, upregulated in early OPSCC when compared with controls, were found in both PPI clusters and among the top six IPA networks. Out of the 42 downregulated S-plot proteins presented in the top six IPA networks, complement component C9 (C9), ficolin-3 (FCN3) and C4b-binding protein alpha chain (C4BPA) had the best *p*(corr), fold change and intensity values, and were also present in the PPI clusters (Fig. 5). In our opinion, together, these five proteins should be further studied as a potential future panel for early OPSCC diagnostics. Being all among S-plot proteins and present in both IPA and PPI networks, they had the best ability to identify cases from controls. CFHR2 is a complement factor found to regulate alternative complement pathway activation.^40^ MYL12A is a myosin regulatory subunit that regulates muscle cell contraction.^25^ This protein has been thought to potentially participate in DNA damage repair,^41^ and upregulation of MYL12A mRNA has been associated with non-small-cell lung carcinoma previously.^42^ C9 is a member of the membrane attack complex, participating in the final component of the complement system.^41^ FCN3 has a role in the activation of the complement pathway through the activation of the lectin pathway.^41^ Downregulation of C9, FCN3 and C4BPA mRNAs has previously been associated with liver cancer.^43, 44^ C4BPA, together with C4BPB, forms a multimeric protein participating in complement activation in the classical pathway.^41^ It is of note that, owing to very small abundances of C4BPB, there is little or no utility for this protein, as it will be hard to detect it reliably with classical clinical chemistry settings. However, C4BPA has all the characteristics of being clinically useful due to good abundance in serum samples, high confidence of identification, good fold change and statistical significance (Table 1 and Supplementary Table 2).

The ratio between upregulated and downregulated proteins and the networks in which these proteins were participating made us hypothesise that in the case of early-stage OPSCCs, the main reason for the change in serum proteome could be a tumour-specific response in the host system, not necessarily proteins originating from the actual tumour. When comparing our results with earlier serum proteomics studies on cancer patients, we discovered that 11 proteins out of the 152 quantified proteins in OPSCC serum were also expressed in the serum of patients with pancreatic cancer and 47 proteins were expressed in the serum of oral cavity squamous cell carcinoma (OSCC).^45, 46^ This finding indicates that changes in the levels of some serum proteins most likely reflect a general response to cancer, with still the largest part being specific to the disease. Even though the networks and functions of the proteins with altered expression levels in OPSCC were quite generalised to cancer, the protein combinations seem to be unique. Interestingly, the differences between OSCC and OPSCC, although smaller than in comparison to pancreatic cancer, were significant. Although cases in the current study represented early tumours, whereas tumours in the OSCC study were of all TNM stages,^46^ it is likely that this significant difference in the protein expression profiles is also due to tumour-specific changes in serum. In addition to these possible changes due to histological and anatomical differences between OPSCC and OSCC, another possible reason for the OSCC/OPSCC difference is the viral origin in half of the OPSCC tumours studied.^8^ The role of HPV in tongue cancers is not established.

When serum samples of patients with p16-positive and p16-negative tumours were compared with each other, 24 proteins were differently expressed in the two groups. S-plot proteins resulting from comparing each group with healthy controls were almost exclusively shared between the two groups, although the fold changes of the proteins’ expressions varied. IPA canonical pathways and networks and PPI network analyses were created separately for p16-negative and p16-positive early-stage OPSCCs versus control data. The majority of the interacting proteins were shared by both groups, as expected, as all the cases represent early-stage OPSCC. Some minor differences in protein interactions segregating the two groups were discovered. For example, a network haematological disease, haematological system development and function and organismal functions were only present in the IPA networks of the p16-positive group. All in all, based on serum proteomics, p16-positive and p16-negative early-stage OPSCCs seemed to be mostly similar, although some specific proteins, networks and PPIs were found.

These results strengthen the current knowledge of OPSCC being a disease with versatile altering events in protein expression levels, and further the knowledge in associating networks and interactions. Most probably, the changes seen in serum protein levels reflect the general host response, tumour-specific host response and leaking of tumour-specific proteins into the bloodstream. The expression levels of 96 S-plot proteins were able to reliably distinguish early-stage OPSCCs from healthy controls. Network and PPI analyses provided some additional information of the proteins, with the ability to filter out a smaller set of proteins—putatively representing a potential panel of biomarkers. This is important, as instead of seeking a single protein, the opportunity to form a panel of proteins with both upregulated and downregulated abundancies could serve as a more dependable composition for decision making in future diagnostics. We suggest that the panel of five serum proteins; CFHR2, MYL12A, C9, FCN3 and C4BPA, identified with these methods, might serve as a diagnostic biomarker for early-stage OPSCC.

To conclude, we have demonstrated how serum proteomics is capable of differentiating patients with early-stage OPSCC from healthy controls. This finding has a great potential to improve the early diagnostics of OPSCC. More importantly, the present study and our earlier work will allow us to further delineate differences between different head and neck cancers in terms of their characteristic serum-biomarker profiles. Further screening of the five above-mentioned proteins in a larger cohort of patients would be necessary to establish their value for clinical use.

## Electronic supplementary material

Supplementary Figure 1

Supplementary Figure 2

Supplementary Figure 3

Supplementary Figure 4

Supplementary Figure 5

Supplementary Figure 6

Supplementary Figure 7

Supplementary Figure 8

Supplementary Figure 9

Supplementary Figure 10

Supplementary Figure 11

Supplementary Table 1

Supplementary Table 2

Supplementary Table 3

Supplementary Table 4

Supplementary Table 4

## Availability of data and materials

The mass spectrometry proteomics is available from the ProteomeXchange Consortium via the PRIDE partner repositorywith the data set identifier PXD008445.
