# Supplementary figures and images for: Patients with early-stage oropharyngeal cancer can be identified with label-free serum proteomics
Source: Br J Cancer. 2018 Jul 2;119(2):200–12. doi: 10.1038/s41416-018-0162-2 (PMC6048110; doi:10.1038/s41416-018-0162-2)

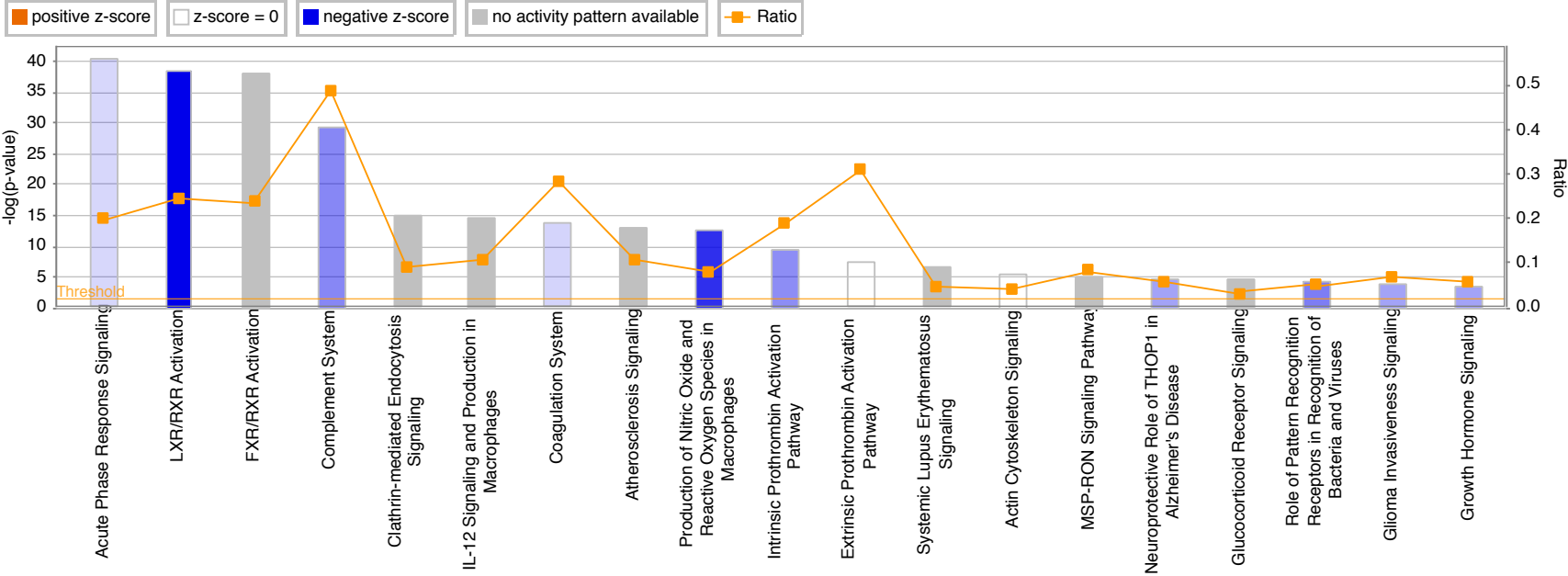

Supplement: Supplementary file 1 — Supplementary Figure 1 [file 41416_2018_162_MOESM1_ESM.pdf]

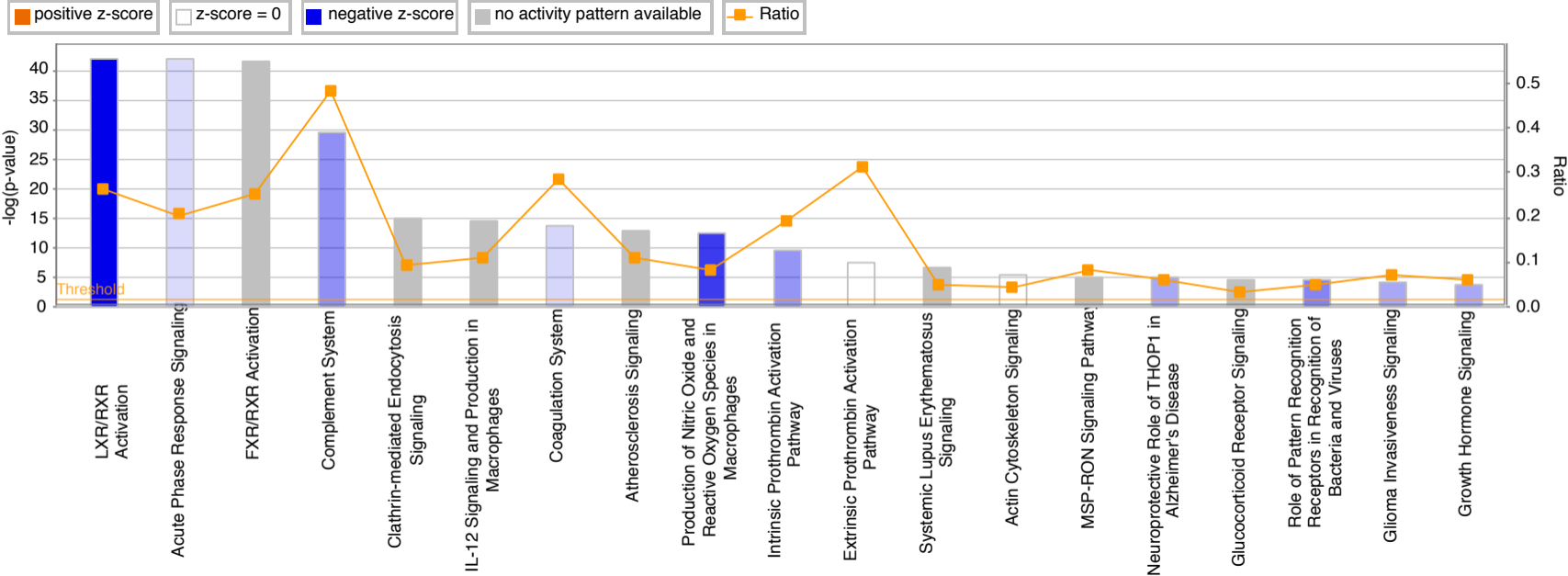

Supplement: Supplementary file 2 — Supplementary Figure 2 [file 41416_2018_162_MOESM2_ESM.pdf]

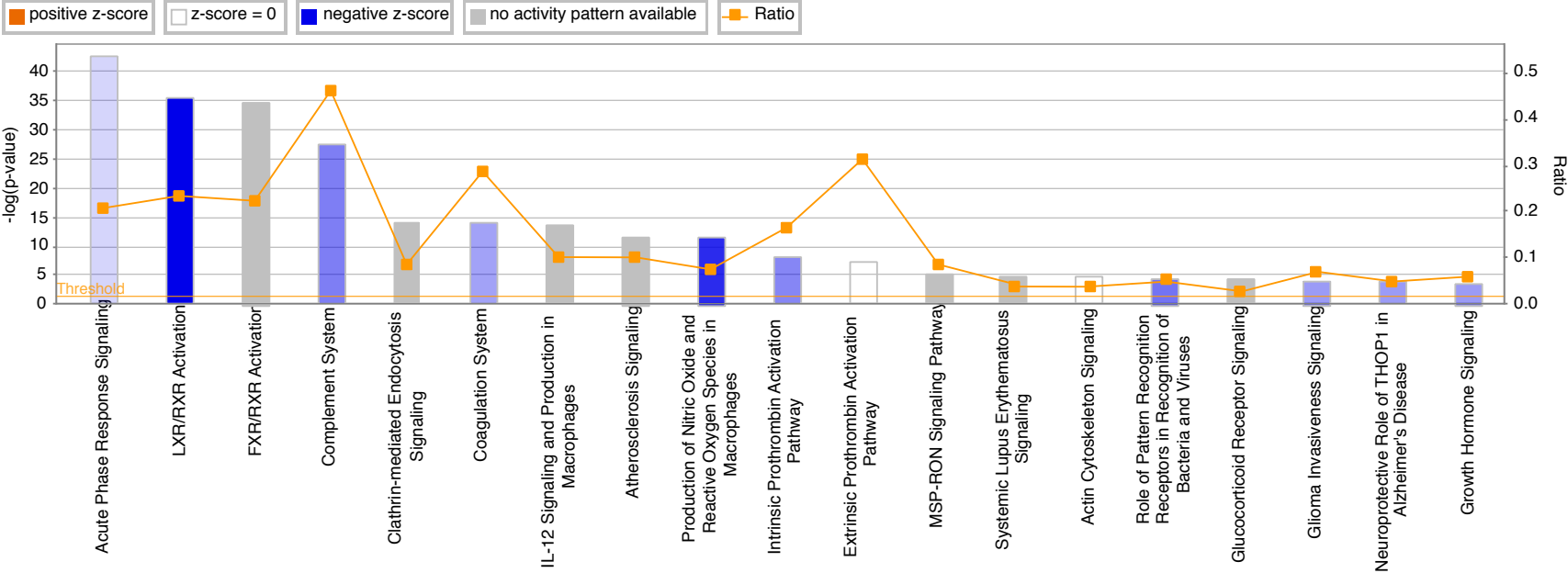

Supplement: Supplementary file 3 — Supplementary Figure 3 [file 41416_2018_162_MOESM3_ESM.pdf]

# Principal Components Analysis

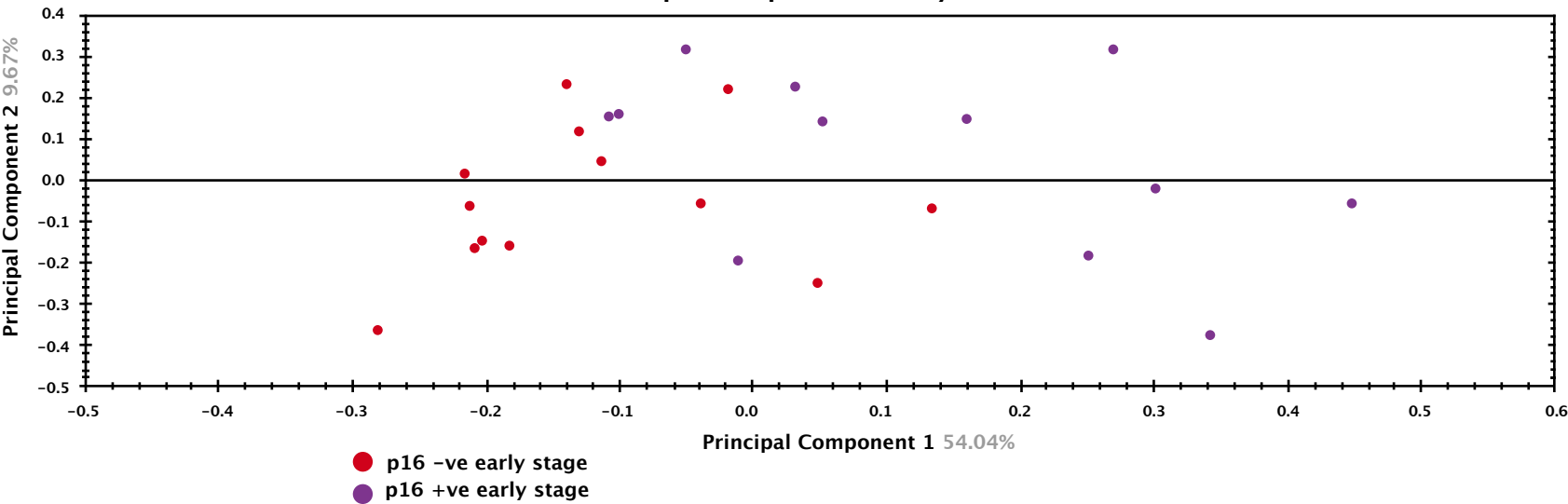

Supplement: Supplementary file 6 — Supplementary Figure 6 [file 41416_2018_162_MOESM6_ESM.pdf]

S-plot (p16+ve early stage OPSCC = -1 versus controls =1)

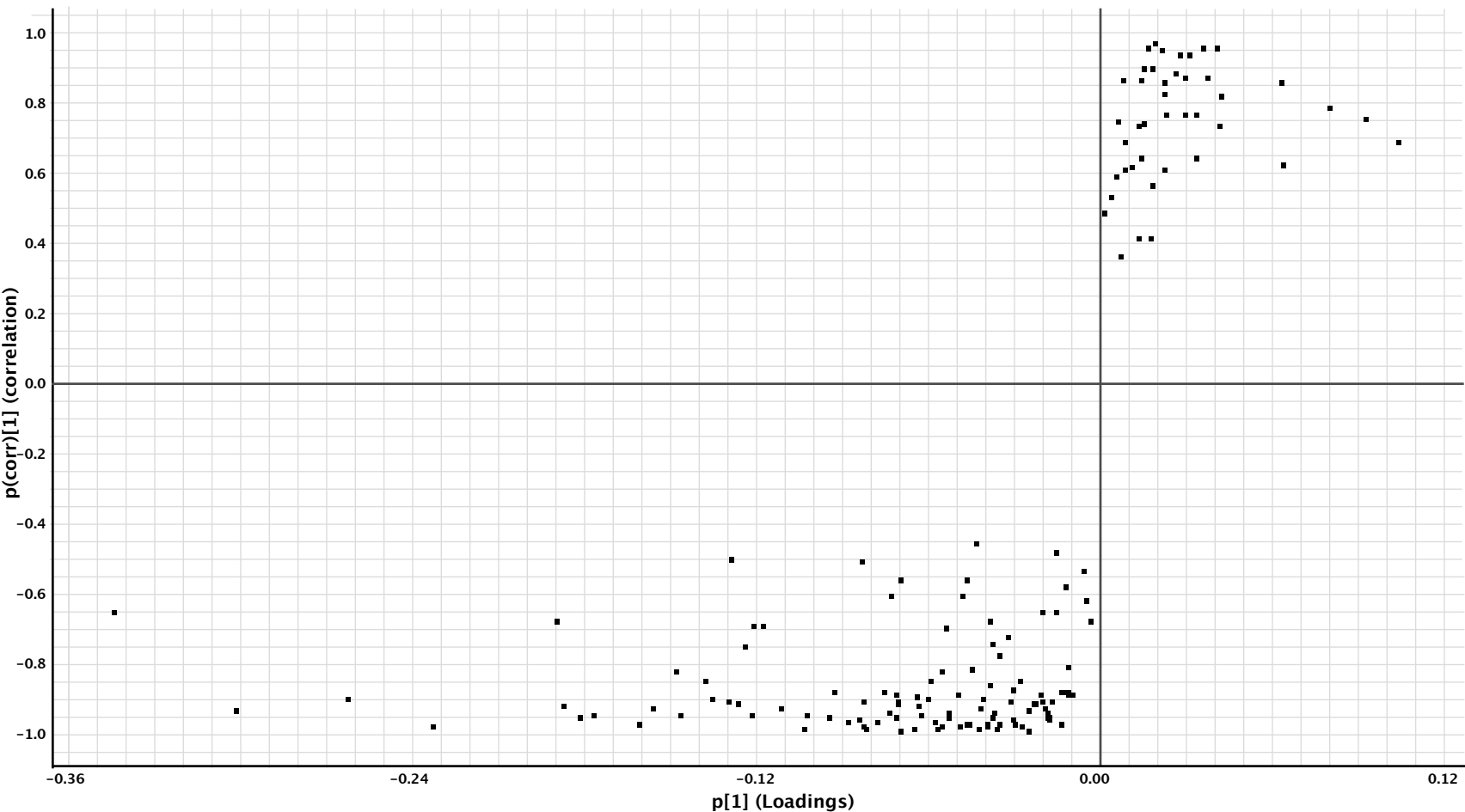

Supplement: Supplementary file 7 — Supplementary Figure 7 [file 41416_2018_162_MOESM7_ESM.pdf]

S-plot (p16-ve early stage OPSCC = -1 versus controls =1)

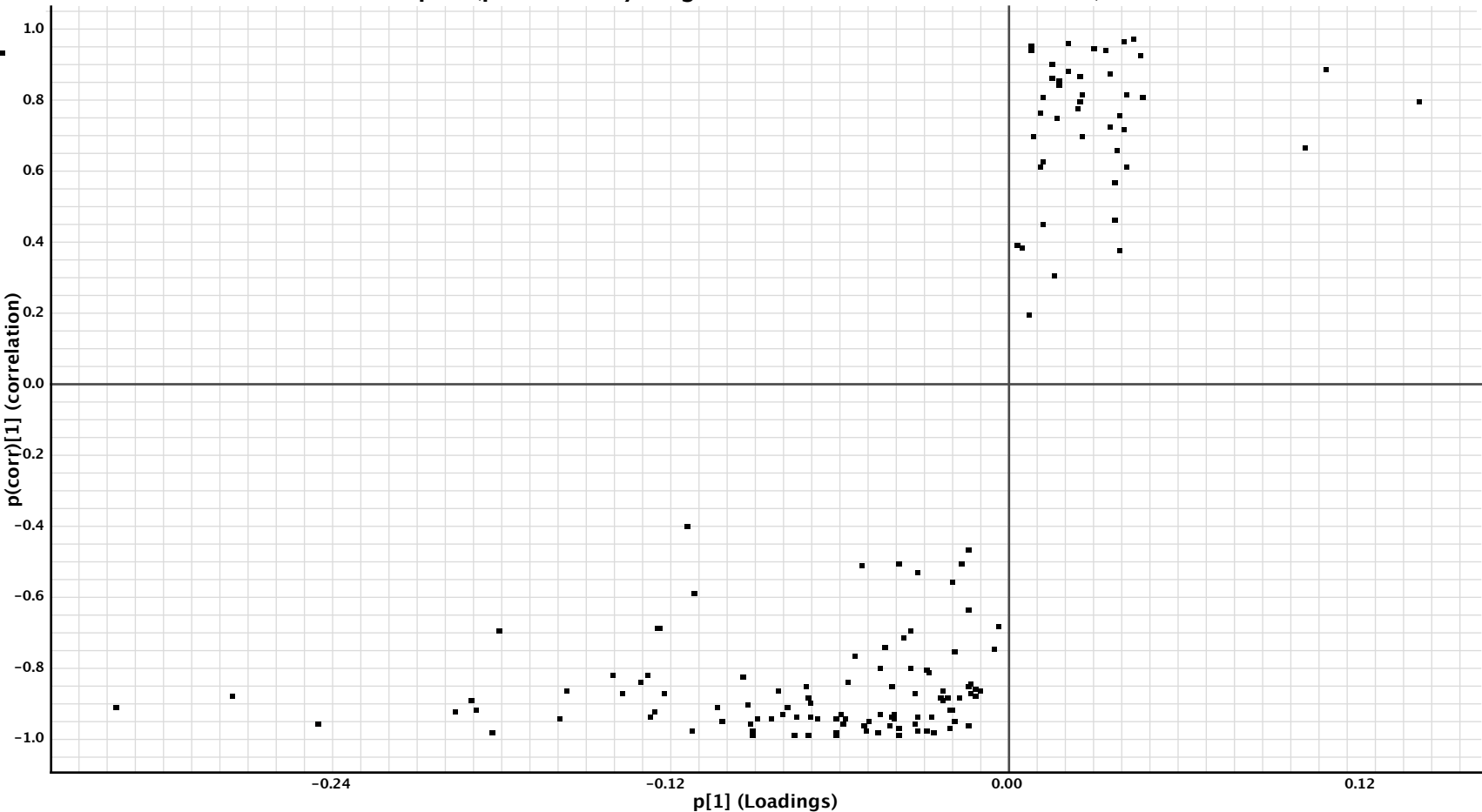

Supplement: Supplementary file 8 — Supplementary Figure 8 [file 41416_2018_162_MOESM8_ESM.pdf]

**p16-ve OPSCC**

**p16+ve OPSCC**

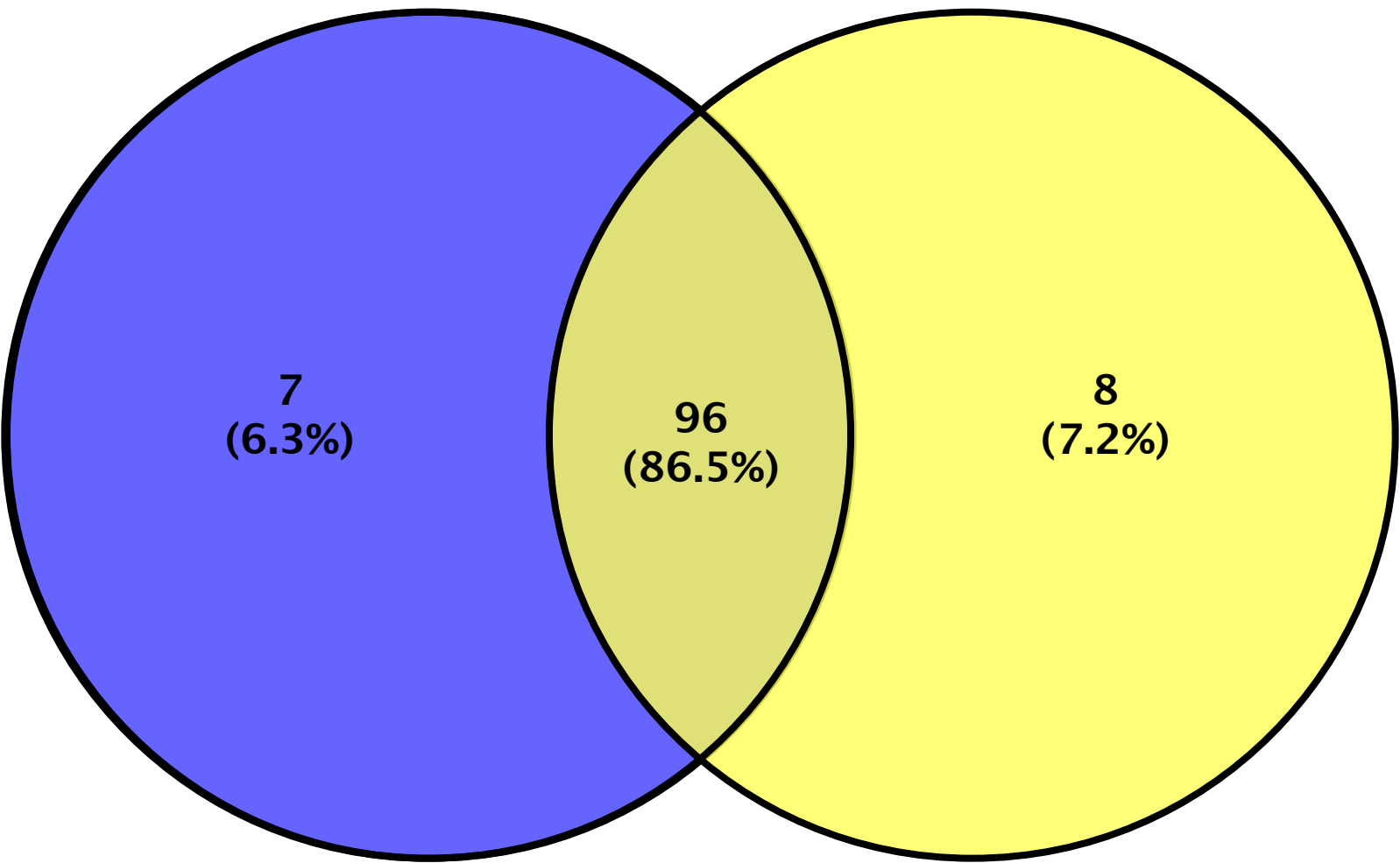

Supplement: Supplementary file 9 — Supplementary Figure 9 [file 41416_2018_162_MOESM9_ESM.pdf]

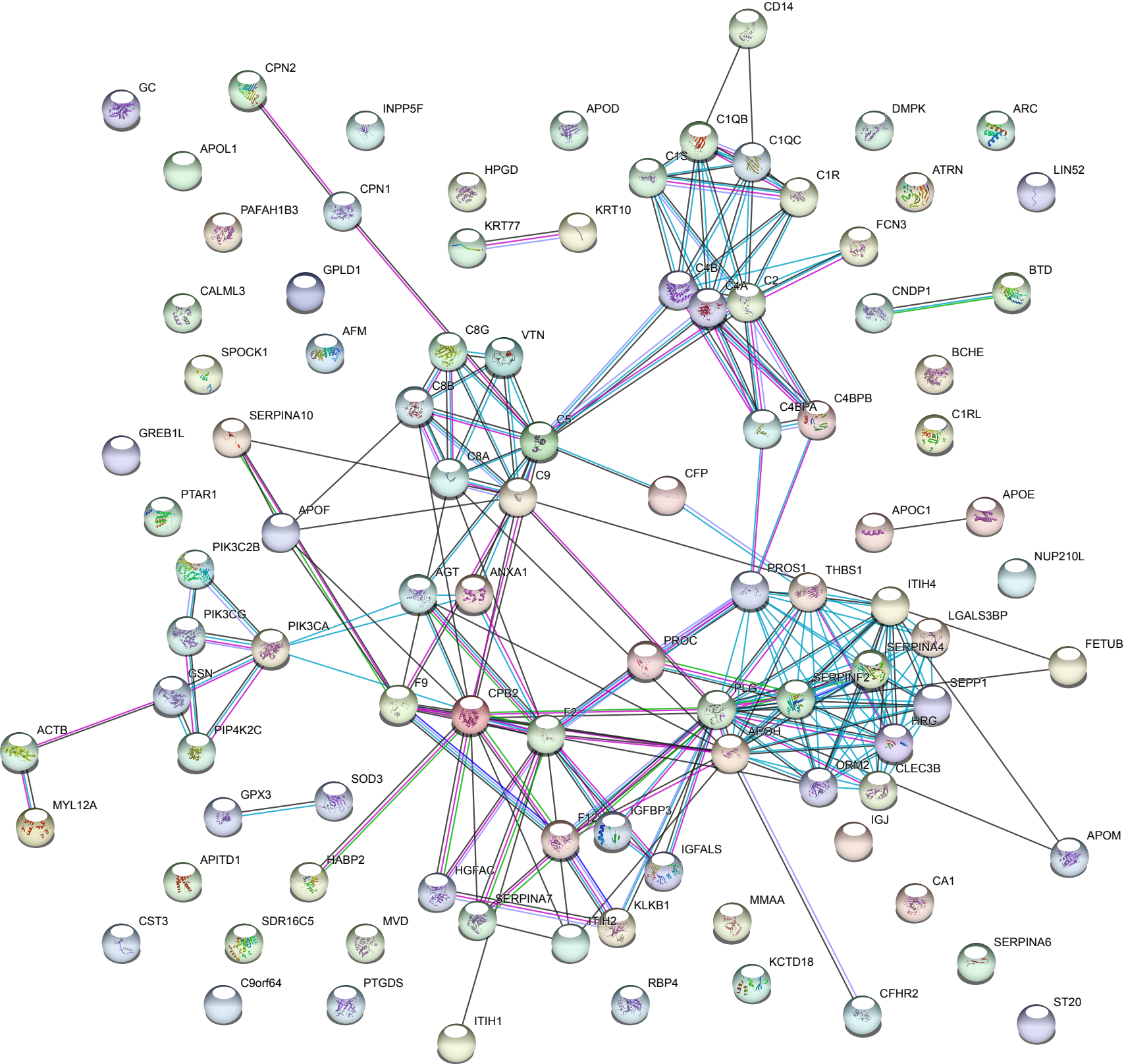

Supplement: Supplementary file 10 — Supplementary Figure 10 [file 41416_2018_162_MOESM10_ESM.pdf]

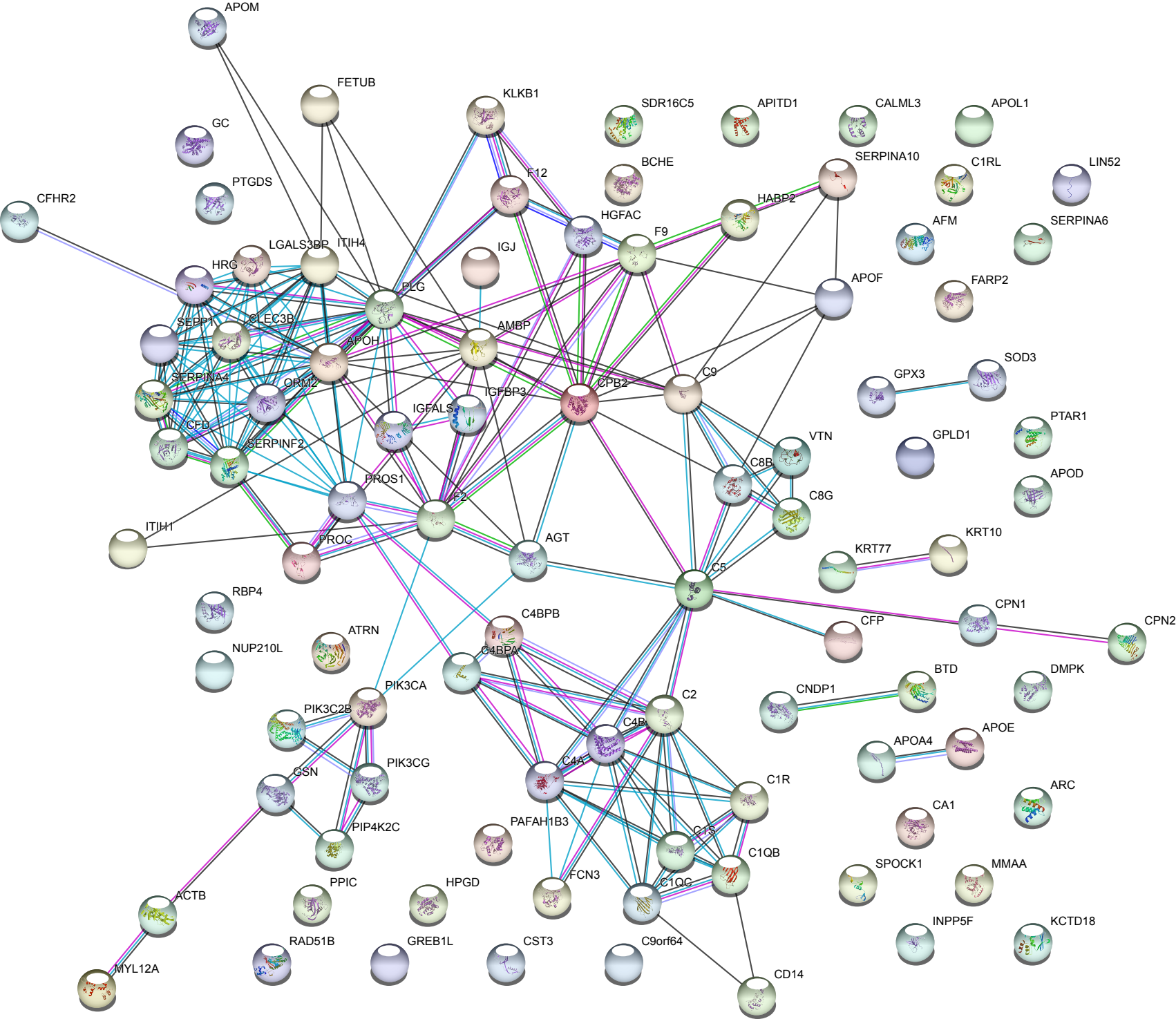

Supplement: Supplementary file 11 — Supplementary Figure 11 [file 41416_2018_162_MOESM11_ESM.pdf]
