# Supplementary material for: Patients with early-stage oropharyngeal cancer can be identified with label-free serum proteomics
Source: Br J Cancer. 2018 Jul 2;119(2):200–12. doi: 10.1038/s41416-018-0162-2 (PMC6048110; doi:10.1038/s41416-018-0162-2)
Supplement: Supplementary file 4 — Supplementary Figure 4 [file 41416_2018_162_MOESM4_ESM.pdf]

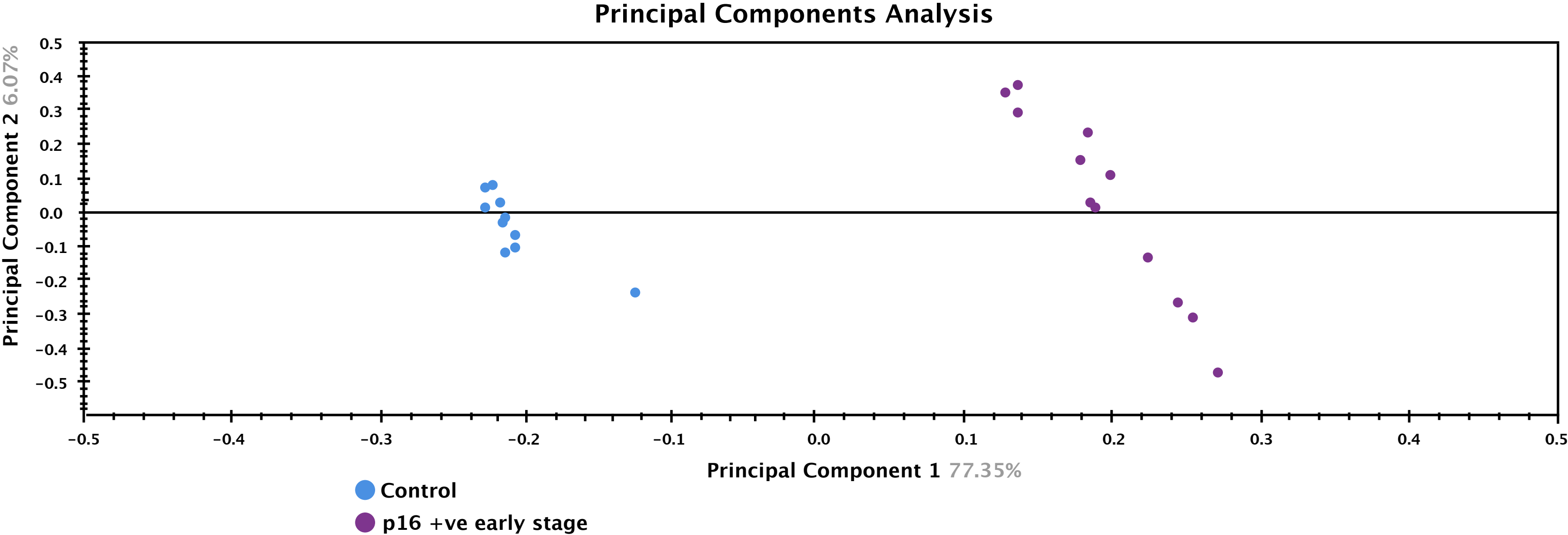
Principal Components Analysis
Principal Component 2 6.07%
0.5
0.4
0.3
0.2
0.1
0.0
−0.1
−0.2
−0.3
−0.4
−0.5
−0.5
−0.4
−0.3
−0.2
−0.1
0.0
0.1
0.2
0.3
0.4
0.5
Principal Component 1 77.35%
Control
p16 +ve early stage
