# Supplementary material for: Patients with early-stage oropharyngeal cancer can be identified with label-free serum proteomics
Source: Br J Cancer. 2018 Jul 2;119(2):200–12. doi: 10.1038/s41416-018-0162-2 (PMC6048110; doi:10.1038/s41416-018-0162-2)
Supplement: Supplementary file 16 — Supplementary Table 4 [file 41416_2018_162_MOESM16_ESM.docx]

**Supplementary Table 1. Clinical parameters** of tumour samples.

**Supplementary Table 2.** 152 proteins separating stage I-II early OPSCC’s from healthy controls’ serum, with ANOVA p-value <0.05.

**Supplementary Table 3. IPA** networks upregulated for stage I-II OPSCC vs controls, p16-positive stage I-II OPSCC vs controls, and p16-negative stage I-II OPSCC vs controls. Molecules in bold indicate focus molecules, downregulated in the tumour compared to controls. Molecules in bold-italic indicate focus molecules upregulated in the tumour compared to controls.

**Supplementary Table 4. S-plot** protein lists for p16+ve stage I-II OPSCC vs controls, and p16-ve stage I-II OPSCC vs controls.

**Supplementary Figure 1.** **IPA Canonical pathways** from the quantified proteins with ANOVA p-value <0.05. All early-stage OPSCCs versus controls.

**Supplementary Figure 2. IPA Canonical pathways** from the quantified proteins with ANOVA p-value <0.05. p16-positive early-stage OPSCCs versus controls.

**Supplementary Figure 3. IPA Canonical pathways** from the quantified proteins with ANOVA p-value <0.05. p16-negative early-stage OPSCCs versus controls.

**Supplementary Figure 4. PCA** using serum protein expression data of early-stage p16+ve OPSCC versus controls (two or more unique peptides, ANOVA p-value <0.05)

**Supplementary Figure 5. PCA** using serum protein expression data of early-stage p16-ve OPSCC versus controls (two or more unique peptides, ANOVA p-value <0.05)

**Supplementary Figure 6. PCA** using serum protein expression data of early-stage p16-ve OPSCC versus p16+ve OPSCC (two or more unique peptides, ANOVA p-value <0.05)

**Supplementary Figure 7**. **S-plot** obtained from OPLS-DA regression analysis of the serum protein expressions (p(corr) ±0.80). The proteins downregulated in p16+ve tumour patients’ serum is in the upper right corner, and the upregulated on the lower left.

**Supplementary Figure 8.** **S-plot** obtained from OPLS-DA regression analysis of the serum protein expressions (p(corr) ±0.80). The proteins downregulated in p16-negative tumour patients’ serum is in the upper right corner, and the upregulated on the lower left.

**Supplementary Figure 9. A Venn** diagram of S-plot proteins in p16-positive and p16-negative stage I-II OPSCCs versus control.

**Supplementary Figure 10. PPI** networks of S-plot proteins (p(corr) ±0.80). p16-positive early stage OPSCC versus controls.

**Supplementary Figure 11. PPI** networks of S-plot proteins (p(corr) ±0.80). p16-negative early stage OPSCC versus controls.
